# A declarative model specification system allowing NeuroML to be extended with user-defined component types

**DOI:** 10.1186/1471-2202-13-S1-P42

**Published:** 2012-07-16

**Authors:** Robert Cannon, Padraig Gleeson, Sharon Crook, R Angus Silver

**Affiliations:** 1 Textensor Limited, Edinburgh, UK; 2 Dept. of Neuroscience, Physiology and Pharmacology, University College London, London, UK; 3 School of Mathematical and Statistical Sciences, School of Life Sciences, Arizona State University, Arizona, USA

## 

Methods for storing and sharing biological models tend to focus on directly encoding a model and its equations. However, many models share the same equations, subject only to differences in parameter values or the number of instances of particular processes. We have therefore developed a mechanism within NeuroML[1] to express the common structural and mathematical features of a family of models separately from the parameter values that define a particular member of the family. Such specifications are important because they correspond closely to the mathematical understanding that computational scientists have for the modeled system. Supporting this conceptual level separately from the models themselves allows particular instances of models to be expressed more concisely and makes the commonalities among models explicit. It also facilitates a wide range of model related processing including systematic comparison, model composition, and sensitivity analysis.

The resulting system, known as LEMS (Low Entropy Model Specification), makes it possible to retro-fit most of the existing NeuroML version 1 definitions with domain independent definitions constructed from LEMS elements. This preserves the benefits of the existing semantically rich high-level concepts in NeuroML while providing the ability to add new concepts at the user level rather than requiring changes to the language. Simulator developers have a choice between directly supporting the library of core types, or supporting the underlying LEMS definitions. These options are not exclusive: a tool could provide hard-coded support for certain key components such as ion channels, and fall back to a generic (and probably less efficient) implementation for new component definitions.

Component type definitions in LEMS consist of three main parts: a declaration of the static properties needed to define a particular type of model; specification of the dynamics of the component; and specification of the component's structure. The static definition includes parameters types, component nesting, and references between components. The dynamics contains the equations that govern the behavior of an instance of the model. The structure definition specifies how the static properties should be interpreted when a component is used. This may be as simple as creating a single instance of each of the state variables, but may also involve instantiating other components or connecting them together as in, for example, component types that express network structure or connectivity.

As well as an extensive library of component types, and example models, NeuroML2/LEMS contains a reference interpreter that can run any LEMS model. Although initial development has been driven by the need to support the requirements of the NeuroML community, the resulting language only includes mathematical and structural elements and should be applicable to other model specification tasks.
